# Prdm Proto-Oncogene Transcription Factor Family Expression and Interaction with the Notch-Hes Pathway in Mouse Neurogenesis

**DOI:** 10.1371/journal.pone.0003859

**Published:** 2008-12-03

**Authors:** Emi Kinameri, Takashi Inoue, Jun Aruga, Itaru Imayoshi, Ryoichiro Kageyama, Tomomi Shimogori, Adrian W. Moore

**Affiliations:** 1 Molecular Neuropathology Group, RIKEN Brain Science Institute, Wako, Saitama, Japan; 2 Laboratory for Behavioral and Developmental Disorders, RIKEN Brain Science Institute, Wako, Saitama, Japan; 3 Institute for Virus Research, Kyoto University, Sakyo-ku, Kyoto, Japan; 4 Shimogori Research Unit, RIKEN Brain Science Institute, Wako, Saitama, Japan; Temasek Life Sciences Laboratory, Singapore

## Abstract

**Background:**

Establishment and maintenance of a functional central nervous system (CNS) requires a highly orchestrated process of neural progenitor cell proliferation, cell cycle exit, and differentiation. An evolutionary conserved program consisting of Notch signalling mediated by basic Helix-Loop-Helix (bHLH) transcription factor activity is necessary for both the maintenance of neural progenitor cell character and the progression of neurogenesis; however, additional players in mammalian CNS neural specification remain largely unknown. In *Drosophila* we recently characterized Hamlet, a transcription factor that mediates Notch signalling and neural cell fate.

**Methodology/Principal Findings:**

Hamlet is a member of the Prdm (PRDI-BF1 and RIZ homology domain containing) proto-oncogene transcription factor family, and in this study we report that multiple genes in the *Prdm* family (*Prdm6*, *8*, *12*, *13* and *16*) are expressed in the developing mouse CNS in a spatially and temporally restricted manner. In developing spinal cord *Prdm8, 12* and *13* are expressed in precise neuronal progenitor zones suggesting that they may specify discrete neuronal subtypes. In developing telencephalon *Prdm12* and *16* are expressed in the ventricular zone in a lateral to medial graded manner, and *Prdm8* is expressed in a complementary domain in postmitotic neurons. In postnatal brain *Prdm8* additionally shows restricted expression in cortical layers 2/3 and 4, the hippocampus, and the amygdala. To further elucidate roles of *Prdm8* and *16* in the developing telencephalon we analyzed the relationship between these factors and the bHLH Hes (Hairy and enhancer of split homolog) effectors of Notch signalling. In *Hes* null telencephalon neural differentiation is enhanced, *Prdm8* expression is upregulated, and *Prdm16* expression is downregulated; conversely *in utero* electroporation of *Hes1* into the developing telencephalon upregulates *Prdm16* expression.

**Conclusions/Significance:**

Our data demonstrate that *Prdm* genes are regulated by the Notch-Hes pathway and represent strong candidates to control neural class specification and the sequential progression of mammalian CNS neurogenesis.

## Introduction

The nervous system of mammals contains a large number of neurons in a diverse array of neuron classes. Transcription factors play central roles in generating this complexity by controlling neural progenitor cell proliferation, patterning, and defining neuron fate [1], [2]. For example, it is well established that an evolutionary conserved basic Helix-Loop-Helix (bHLH) transcription factor cascade downstream of Notch signalling is necessary for both the maintenance of neural progenitor cell character and the progression of neurogenesis. High Notch activity maintains neural progenitors through an effector pathway consisting of the bHLH Hairy and enhancer of split homologue transcription factors Hes1 and Hes5. Notch upregulates the Hes factors that then function as DNA-binding repressors and antagonize the expression of proneural bHLH genes [3]. Hence, low Notch activity reduces Hes activity and leads to upregulation of proneural bHLH factors such as Ngn2 (Neurogenin2) and Mash1 (Mammalian achaete-scute homolog1); these factors then repress neural progenitor cell maintenance and promote neuron differentiation [4].

**Figure 1:**
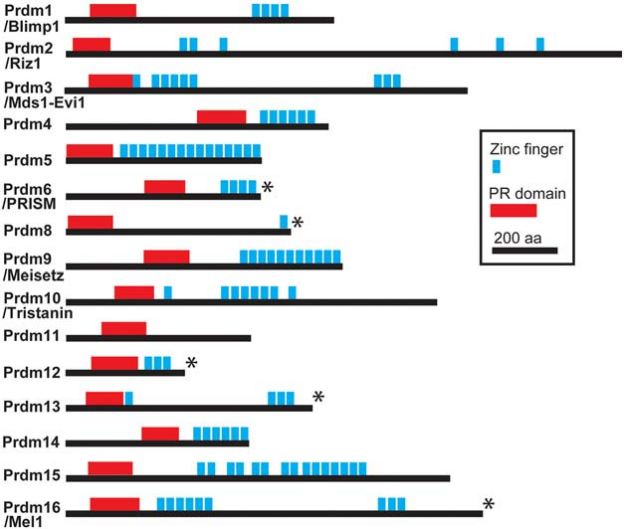
The domain structure of the mouse Prdm1–16 proteins. Illustrations of the protein domain structure of the Prdm family members 1–16. These illustrations are drawn to scale based on the sequence analysis shown in Table S1. Zinc fingers are represented by blue boxes and the PR domain by a red box. Those proteins for which a nervous system specific expression pattern is reported in this study are indicated with asterisks.

Much of our understanding of the mechanisms of Notch and bHLH function in the mammalian central nervous system (CNS) is derived from seminal studies examining neurogenesis in the peripheral nervous system (PNS) of the fruit fly *Drosophila melanogaster* [5]. In the fly we recently identified Hamlet, a transcription factor as acting to instruct neuron and glial cell fate and mediating Notch signalling in a neural lineage-specific manner [6], [7]. Hamlet is a member of the relatively uncharacterized transcription factor family known as the Prdm (PRDI-BF1 and RIZ homology domain containing) family [8]. Prdm family members are characterized by an N-terminal PR domain, and in addition all but one (Prdm11) contain zinc fingers (Fig. 1). The PR domain is 20–30% identical to the SET (Su(var)3-9, Enhancer-of-zeste, and Trithorax) domain, a histone methyltransferase catalytic module [9].

We hypothesized that the *Prdm* gene family may also significantly participate in the development of the mammalian nervous system as this family meets the molecular criteria to be active in neurogenesis. *Prdm* family members are known to control cell proliferation both in cancer [10]–[15] and in normal development [16]. Furthermore, *Prdm* family members are also used to define cell fate. For example, a great deal of interest has been generated by the abilities of *Prdm16* to control the switch between skeletal muscle and brown fat in mice [17], [18], and *Prdm1* to act as a switch between fast and slow twitch muscle in zebrafish (*Danio rerio*) [19]. In addition, Notch signalling is an essential control mechanism in neurogenesis, and *Prdm* family function in cell fate can occur through mediation of Notch signalling. For example, both *Drosophila hamlet* and its *Caenorhabditis elegans* homologue *EGL-43* mediate Notch-controlled cell fate decisions [7], [20], [21].

Roles in nervous system development have already been demonstrated or suggested for a few members of the *Prdm* family. Both *hamlet* and *EGL-43* are required for sensory neuron differentiation [6], [22]. Furthermore, *Prdm3* (*Mds1/Evi1*), the mouse homologue of *hamlet*, is also expressed in the PNS within the developing cranial and dorsal root ganglia [23], and knockout of *Prdm3* in mice leads to nervous system hypoplasia [24]. *prdm1* (*blimp1*) is expressed in sensory neuron precursors in both zebrafish [25] and *Drosophila* [26]. In zebrafish *prdm1* expression at the edge of the neural plate specifies the precursor cells competent to form primary sensory neurons [25], [27]. In these cells *prdm1* functions within the context of a Notch-bHLH pathway since sensory neurogenesis also requires downregulation of Notch signalling and subsequent induction of *ngn1* [28].

In this study we present evidence for the function of multiple relatively uncharacterized *Prdm* gene family members during mammalian neurogenesis. By employing mRNA *in situ* hybridization (ISH) analysis we show that several *Prdm* family members are expressed in spatially restricted and related domains of neuronal progenitors in the developing CNS consistent with a role in neural class specification. In addition, we find that a subset of *Prdm* family members remain expressed in the postnatal brain. Furthermore, by analyzing *Hes* loss- and gain-of-function embryos, we demonstrate that *Prdm* family gene expression in the developing telencephalon is controlled by the Notch-Hes pathway and regulated during the sequential progression of neurogenesis. We suggest that the genes of the *Prdm* family represent strong new candidates to function in neural progenitor cell proliferation and neural differentiation in the mammalian CNS.

## Results

### *Prdm5–16* expression in midgestation mouse embryos

Fifteen mouse members of the *Prdm* family (Fig. 1, Table S1) were identified from the National Center for Biology Information (NCBI) http://www.ncbi.nlm.nih.gov/ and Mouse Genome Informatics (MGI) http://www.informatics.jax.org/ databases. The expression patterns of *Prdm1*–*4* have been investigated in detail; of these, *Prdm1, 3* and *4* are expressed in the nervous system [23], [29], [30] (data not shown). *Prdm2* expression has not been reported in the CNS and our preliminary studies detected no CNS-specific expression (data not shown).

We designed two or three independent, gene-specific primer pairs for *Prdm5*–*16*. Using these primers we carried out reverse transcription polymerase chain reaction (RT-PCR) on total mRNA isolated from stage embryonic (E) day 11.5 (whole embryo) and 13.5 (head only) tissue. In both samples we detected expression of all *Prdm5*–*16* genes (data not shown). Furthermore, we confirmed that we had amplified cDNA from the correct predicted gene by sequencing the RT-PCR-generated amplicons from each primer pair.

### *Prdm8, 12* and *13* show restricted nervous system expression from early embryogenesis

**Figure 2:**
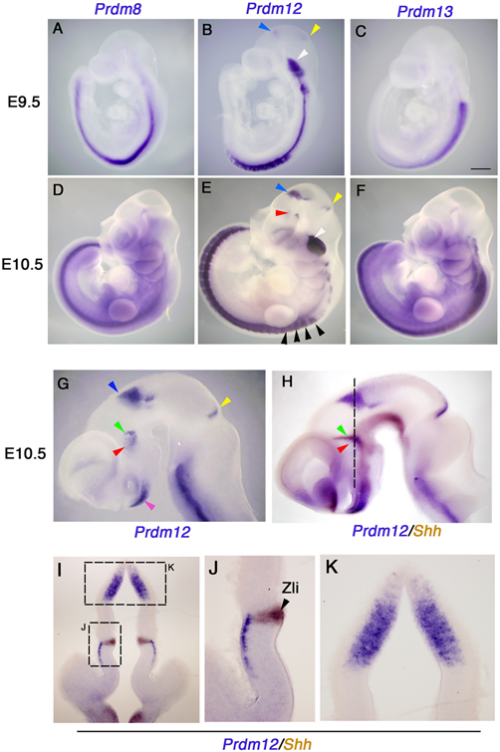
Whole mount ISH analysis of the *Prdm* gene family at E9.5 and E10.5. The spatial distribution of *Prdm8, 12*, and *13* are shown by WISH at E9.5 (A–C) and E10.5 (D–F) in mouse. Strong expression of *Prdm8* (A, D) and *Prdm13* (C, F) is observed in spinal cord at E9.5 and E10.5. Strong expression of *Prdm12* in spinal cord and in cranial ganglia (white arrowhead) and weak expression in caudal diencephalon and midbrain (blue and yellow arrowheads) is observed at E9.5 (B). (E) At E10.5, additional expression of *Prdm12* in the rostral brain is observed in p3 (red arrowhead), and stronger expression in p1 (blue arrowhead) and the midbrain (yellow arrowhead). In addition *Prdm12* is expressed in the dorsal root ganglia (black arrowheads) and cranial ganglia (white arrowhead). (G–I) WISH of the dissected brain at E10.5 is shown, lateral is to the left. *Prdm12* is expressed in the ventral hindbrain, p3 (red and green arrowheads), p1 (blue arrowheads), midbrain (yellow arrowhead), and hypothalamus (pink arrowheads) (G). Two-colour WISH for *Prdm12* (blue) and *Shh* (brown) in Zli (zona limitans intrathalamica) shows the spatial relationships of the expression domains of these genes (red and green arrowheads) (H). A section taken at the plane illustrated with a dashed line in panel H is shown in panel I. In addition, magnified regions from panel I, highlighted by dashed boxes, are shown in panel J and K. Bar in C, 500 µm (A–C), 700 µm (D–F), 400 µm (G–H), 200 µm (I), and 100 µm (J, K).

To examine the expression of *Prdm5*–*16* in detail, we carried out whole mount *in situ* hybridization (WISH) at E9.5 and E10.5. Three *Prdm* genes (*8*, *12* and *13*) showed spatially restricted expression in nervous system tissue (Fig. 2). *Prdm8* and *13* showed specific expression in spinal cord at E9.5 when neurogenesis starts (Fig. 2A, C), and maintained their expression in this tissue at E10.5 (Fig. 2D, F).

*Prdm12* was also expressed in the spinal cord at E9.5. In addition, weak expression of *Prdm12* was observed in the caudal forebrain and midbrain (Fig. 2B, blue and yellow arrowheads). At E10.5, these two expression domains of *Prdm12* in forebrain and midbrain became stronger (Fig. 2E, red, blue and yellow arrowheads, respectively). For better visibility, we next dissected out the brain at E10.5 to analyze detailed expression of *Prdm12* (Fig. 2G). *Prdm12* was expressed in several regions of the diencephalon, p3 (Fig. 2G, red and green arrowheads), p1 (Fig. 2G, blue arrowhead), hypothalamus (Fig. 2G, pink arrowhead), and a small dorsal region in the midbrain (Fig. 2G, yellow arrowhead). To obtain precise spatial information about the *Prdm12* expression domain in the diencephalon we performed two-colour ISH to compare *Prdm12* expression with *Shh* (sonic hedgehog). *Shh* marks the Zli (zona limitans intrathalamica), the definitive border of p2 and p3 (Fig. 2H–K) [31]. Our analysis clearly revealed that *Prdm12* was expressed in postmitotic neurons adjacent to, but not overlapping, the Zli (Fig 2I, J). In addition, *Prdm12* was expressed in the p1 region in the diencephalic VZ but was excluded from the dorsal midline (Fig. 2I, K).

In addition to CNS expression, we also detected *Prdm12* expression in the PNS. *Prdm12* was expressed in a repeated pattern lateral to the spinal cord in the dorsal root ganglia (DRG) (Fig. 2E, black arrowheads) and in the head region in the cranial ganglia (Fig. 2B, E, white arrowheads).

### *Prdm8, 12* and *13* family members are expressed in interrelated domains along the dorsal-ventral axis of the spinal cord

**Figure 3:**
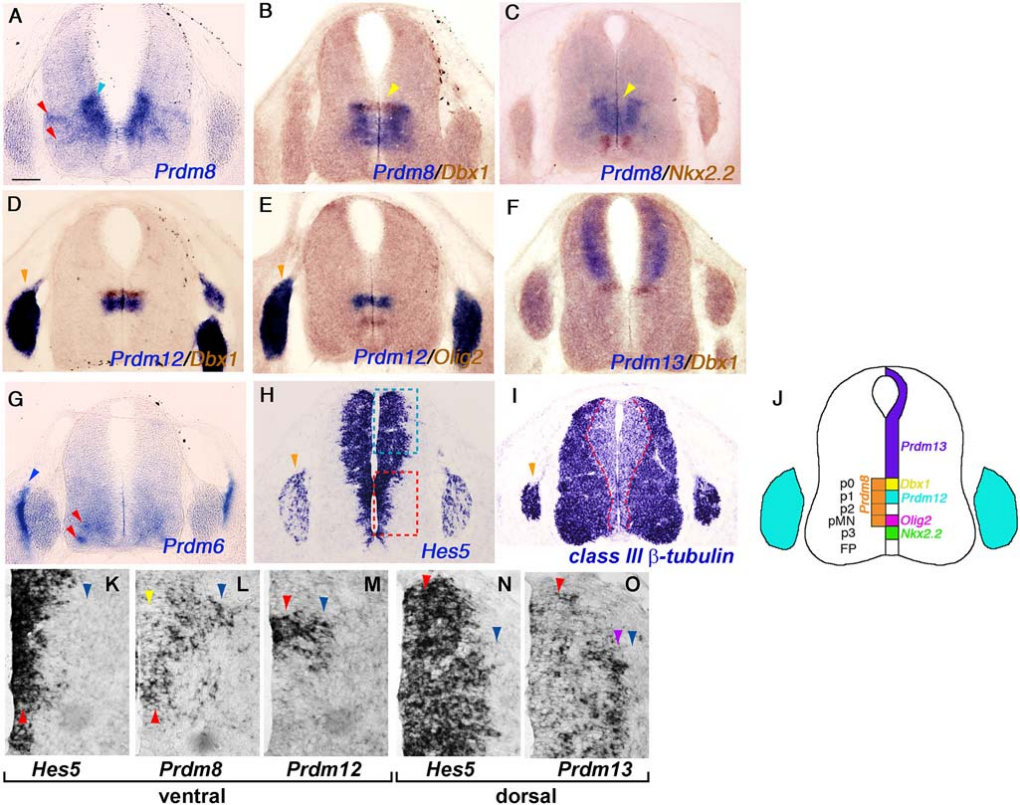
Spatially restricted expression of *Prdm* family members in the spinal cord. Spatial expression of *Prdm* gene family members is shown by ISH in transverse spinal cord cervical sections at E11.5. (A) *Prdm8* has strong expression in the ventral VZ (blue arrowhead) and some postmitotic neurons (red arrowheads). (B, C) Two-colour ISH for *Prdm8* (blue) and *Dbx1* or *Nkx2.2* (brown) in spinal cord progenitor regions shows the dorsal and ventral expression limits of *Prdm8*. (D, E) Two-colour ISH for *Prdm12* (blue) and *Dbx1* or *Olig2* (brown) demonstrate that the *Prdm12* expression domain is restricted to p1. Strong expression in the DRG is also observed (orange arrowhead). (F) Expression of *Prdm13* (blue) is restricted to the dorsal half of spinal cord and its ventral limit is the *Dbx1* (brown) expressing p0 progenitor domain. (G) Expression of *Prdm6* is observed in ventrally located postmitotic neurons (red arrowheads) and also is in putative sclerotome (blue arrowhead). (H) *Hes5* marks the ventricular zone and a small population of precursor cells in the DRG (orange arrowhead). (I) *class III ß-tubulin* marks postmitotic differentiating neurons in the mantle zone (inside the red dashed lines) and DRG (orange arrowhead). (J) A summary cartoon illustrating which progenitor domains express the transcription factors shown in panels A–F. Bar in A 100 µm (A–I). (K–O) A comparison of *Hes5* and *Prdm* family gene expression; each panel shows a magnified region of the spinal cord as boxed in red (K–M) and blue (N, O) in panel H. In the ventral spinal cord *Hes5* (K) expression marks the VZ (red arrowhead) and is not expressed in postmitotic neurons (blue arrowhead); on the other hand, *Prdm8* (L) and *Prdm12* (M) are expressed in both the VZ (red arrowhead) and postmitotic region (blue arrowhead). *Prdm8* expression in the *Dbx1* zone is low in the ventricular zone where it overlaps *Dbx1* and stronger in the mantle zone a where it expands beyond the extent of *Dbx1* expression (yellow arrowheads in B, C, L). In the dorsal spinal cord *Hes5* expression (N) and *Prdm13* expression (O) overlap. *Prdm13* expression is weak in the proximal VZ (red arrowhead) and strong (purple arrowhead) at the interface between the VZ and the mantle zone (blue arrowhead).

To understand the detailed expression pattern of the *Prdm* gene family members in the spinal cord we performed further ISH on sections (Fig. 3). In the ventral neural tube distinct classes of motor- and interneurons are derived from distinct VZ progenitor cell populations. Each progenitor cell population is defined by the expression of subsets of homeodomain (HD) transcription factors [32]. We used two-colour ISH at the cervical level to map the expression domains of *Prdm8, 12* and *13* in the VZ relative to previously described HD factors.

The dorsal limit of the *Prdm8* (Fig. 3A–C) expression domain was identical to that of *Dbx1* (*Developing brain homeobox 1*), which marks the neuronal progenitor domain p0 (Fig. 3B, J) [33]. The *Prdm8* expression domain extended to a ventral limit overlapping with that of *Olig2* (*Oligodendrocyte transcription factor 2*) (data not shown) and was exclusive to the expression domain of *Nkx2.2* (*NK2 transcription factor related, locus 2*) (Fig. 3C, J) [34], [35]. Hence, the *Prdm8* expression domain encompassed the VZ of progenitor regions p0, p1, p2 and pMN (Fig. 3J). *Prdm12* expression (Fig. 3D–E) had a dorsal limit at the *Dbx1* expression domain (Fig. 3D) and a ventral limit significantly dorsal to *Olig2* (Fig. 3E); hence, *Prdm12* was expressed only in the p1 (Fig. 3J). In addition, *Prdm12* was expressed in the DRG (Fig. 3D, E, orange arrowheads). *Prdm13* was localized in the VZ of the dorsal spinal cord with a ventral expression border at the *Dbx1* dorsal limit (Fig. 3F, J).

In the spinal cord expression of *Hes5* marks the proliferating neural precursors in the VZ and *classIII ß-tubulin* marks postmitotic neurons in the mantle zone (Fig. 3H, I, K, N). In the ventral spinal cord *Prdm6* was expressed not in the VZ, but solely in a small subset of postmitotic neurons (Fig. 3G). However, both *Prdm12* and *8* had more complex expression patterns encompassing both *Hes5*-positive proliferating VZ cells and adjacent postmitotic cells in the mantle zone (Fig. 3K–M). Furthermore, the localization of *Prdm8* in the VZ was not consistent along the dorso-ventral axis. At the dorsal limit of *Prdm8* expression, where *Dbx1* was also expressed, *Prdm8* was present only weakly in the VZ but much more strongly in the mantle zone distal to the region expressing *Dbx1* (Fig. 3B, C, L, yellow arrowheads). In the dorsal spinal cord *Prdm13* was expressed throughout the VZ but was highest in the cells at the VZ lateral margin (Fig. 3N–O).

### *Prdm8, 12* and *16* label interrelated domains in the developing forebrain

**Figure 4:**
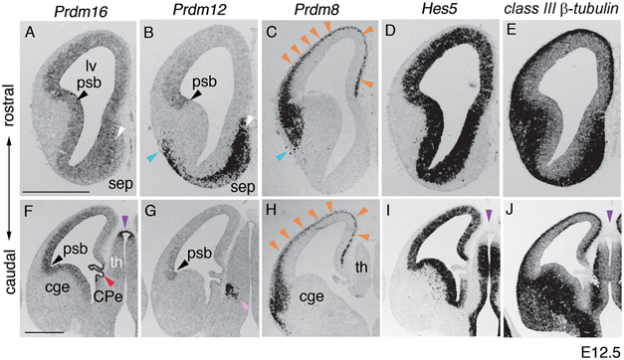
Expression of *Prdm* family members in the developing forebrain. Each panel shows ISH carried out on one hemisphere of either rostral or caudal E12.5 forebrain. (A) *Prdm16* is expressed in the VZ of the dorsal and ventral telencephalon in a lateral/strong to medial/weak gradient. (F) Sections from the caudal part of the brain show expression of *Prdm16* in choroid plexus epithelium (CPe) (red arrowhead) and pretectum (purple arrowhead). The boundary of the pallium and subpallium (psb) is marked by a black arrowhead. (B) *Prdm12* is expressed in lateral telencephalic VZ with a steep lateral/strong to medial/weak gradient from the psb (black arrowhead). It also has expression in postmitotic neurons in the septum (white arrowhead) and pallium (blue arrowhead). (G) Beside the expression of *Prdm12* in the lateral VZ, a small expression domain in the prethalamus (pink arrowhead) is detected. (C, H) *Prdm8* is expressed in the postmitotic neurons of the lateral and dorsal regions of the cortex (orange arrowheads). (D, E, I, J) The progenitor region and the postmitotic region is indicated by *Hes5* (D, I) and *classIII ß-tubulin* (E, J) respectively. Scale bar in A, 250µm (A–E); scale bar in F, 500µm (F–J). Abbreviations: lv, lateral ventricle; psb, pallium-subpallium boundary; sep, septum; cge, caudal ganglionic eminence; th, thalamus; CPe, choroid plexus epithelium.

WISH on embryos isolated at E9.5, E10.5 and E11.5 demonstrated that *Prdm12* was expressed in the brain from E9.5 (Fig. 2) and that *Prdm8* and *16* began to be expressed in the forebrain at E11.5 (data not shown). To analyze brain-specific expression of these factors in more detail we performed section ISH at E12.5 (Fig. 4A–C, F–H). We compared the expression pattern of the *Prdm* genes to those of *Hes5*, which marks proliferating VZ cells, and *classIII ß-tubulin*, which marks postmitotic neurons (Fig. 4D, E, I, J).

At E12.5 *Prdm16* was expressed in the VZ throughout the rostral to caudal telencephalon (Fig. 4A, F). The expression was strongest at the pallium/subpallium boundary (psb) (Fig. 4A, F black arrowhead) and formed a lateral/strong to medial/weak gradient (Fig. 4A, F). *Prdm16* was additionally expressed in the septum (Fig. 4A, white arrowhead), choroid plexus (Fig. 4F, red arrowhead), and pretectum in the diencephalon (Fig. 4F, purple arrowhead). *Prdm12* was expressed in the dorsal telencephalic VZ with a steep lateral/strong to medial/weak gradient from the psb (Fig. 4B, G black arrowhead). *Prdm12* was also expressed in postmitotic neurons in the septum, striatum (Fig. 4B, white and blue arrowheads), and lateral region of the prethalamus (Fig. 4G, pink arrowhead). *Prdm8* was solely expressed in postmitotic neurons in the dorsal telencephalon (Fig. 4C, H, orange arrowheads). In the rostral cortex the lateral edge of the *Prdm8* expression domain was complementary to that of the *Prdm12* expression domain in the pallium (Fig. 4B, C, blue arrowheads).

### *Prdm8* and *12* are expressed in specific populations of neurons in the postnatal brain

To examine later stages of *Prdm* expression we extended our ISH analysis to E16.5 and early postnatal day (P) 6 brains. At E16.5 *Prdm8* continued to be str7ongly expressed in postmitotic neurons in the developing forebrain (data not shown). Furthermore, at E16.5 *Prdm16* was still weakly expressed in the telencephalic VZ; however, *Prdm12* was no longer expressed in this structure (data not shown). At E16.5 both *Prdm16* and *Prdm12* continued to be expressed in the septum (data not shown).

**Figure 5:**
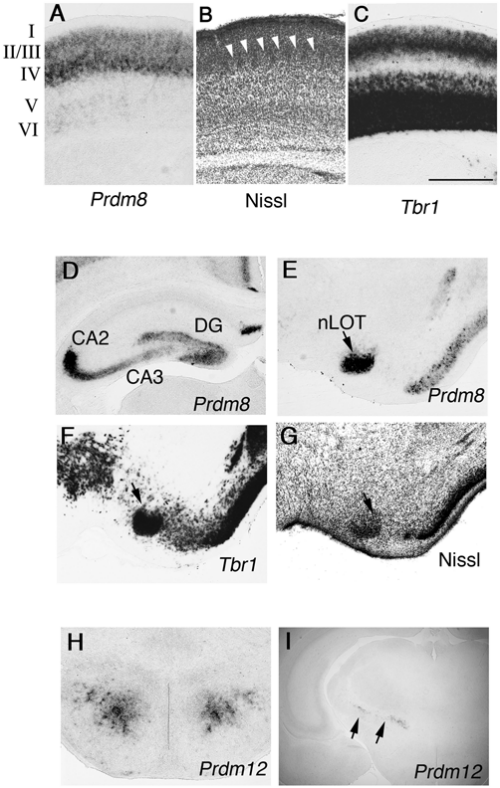
Expression of *Prdm* family genes in the early postnatal brain. Coronal sections through P6 brains, processed to show *Prdm8* expression (A, D, E), *Prdm12* expression (H, I), *Tbr1* expression (C, F) or Nissl (B, G). *Prdm8* is expressed in layer 2/3 and 4 of the neocortex (A). Nissl stain clearly reveals the cortical layers, especially the barrel hollows in the somatosensory cortex in layer 4 (B, arrowheads). Layer 2/3, 5, and 6 are specifically labelled by *Tbr1* (C). Cells expressing *Prdm8* in the hippocampus are concentrated in the dentate gyrus (DG) and the CA2/3 regions (D). At P6, *Prdm8* is expressed specifically in nucleus of lateral olfactory tract (nLOT) (E), which is also revealed by *Tbr1* (F) and Nissl staining (G). *Prdm12* expression in the hypothalamus is restricted in dorsomedial nucleus (H). In the thalamus, expression of *Prdm12* is specific to the dorsal half of the zona incerta (I, arrows). Scale bar in C is 500 µm for A–H and 1.3 mm for I.

At P6 *Prdm8* expression was in a sharply defined lamina pattern in the neocortex (Fig. 5A). Comparison of the *Prdm8* expression domain with Nissl staining and *Tbr1* (*T-box brain gene 1*) [36] expression domains (Fig. 5B, white arrowheads, C) showed that *Prdm8* was expressed in layers 2/3 and 4 (Fig. 5A). *Prdm8*-expressing cells were also scattered in dentate gyrus (DG) and in CA2 and CA3 regions of the pyramidal cell layer in the hippocampus (Fig. 5D). In addition, *Prdm8* was expressed in the nucleus of the lateral olfactory tract (nLOT) (Fig. 5E, arrow) as confirmed by the identical expression of *Tbr1* (Fig. 5F, arrow) and by Nissl staining (Fig. 5G, arrow). The nLOT is connected to the main olfactory bulb and the piriform cortex, and influences nonpheromonal olfactory-guided behaviours, especially feeding [37].

By P6 *Prdm16* was no longer expressed. *Prdm12*, on the other hand, was expressed in specific populations of postmitotic neurons in the hypothalamus where it was restricted to the dorsomedial nucleus (Fig. 5H). *Prdm12* was also expressed in the dorsal half of the zona incerta of the thalamus (Fig. 5I, arrows). Taken together, these results suggest additional roles of *Prdm8* and *12* in differentiated neurons besides their roles in progenitors in the developing CNS.

### *Prdm16* is positively regulated and *Prdm8* negatively regulated by *Hes* activity during telencephalic neurogenesis

Notch signalling is an essential control mechanism to regulate mammalian neurogenesis. We have previously shown that the *Drosophila Prdm* gene *hamlet* is a modifier of Notch signalling during *Drosophila* peripheral neurogenesis [7]. *C. elegans EGL-43* was also demonstrated to be downstream of Notch signalling and to mediate the effect of Notch during vulva formation [20], [21]. Finally, zebrafish *prdm1* is required to enable Notch signalling pathway-mediated specification of sensory neurons [25], [28].

In mammalian cortical neurogenesis high levels of Notch signalling maintain neural progenitors by upregulating the bHLH effectors *Hes1* and *Hes5* [3]. In particular, *Hes1* maintains neural progenitor cell identity and represses proneural genes such as *Ngn2*. These proneural factors drive the progenitor to exit cell cycle and begin neural differentiation [38]; hence, loss of Hes activity leads to upregulation of proneural genes and consequent premature neurogenesis. *Prdm16* expression in the telencephalic ventricular zone overlaps with that of the *Hes* genes (Fig. 4A, D, F, I). Furthermore, the expression of *Prdm8* in early telencephalic development is complementary to that of the *Hes* genes (Fig. 4C, D, H, I). Hence, we examined if the *Hes* effectors of Notch signalling regulate *Prdm* family member gene expression during vertebrate telencephalic neurogenesis.

**Figure 6:**
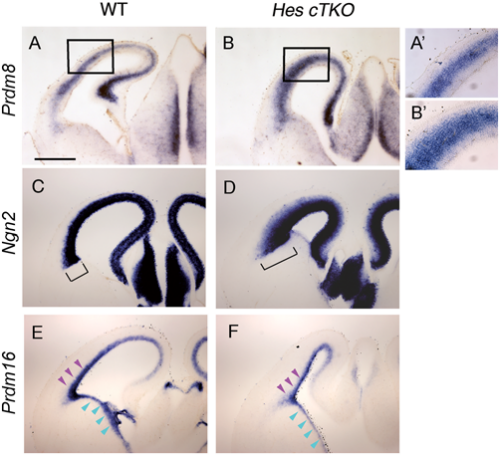
*Prdm8* is upregulated, and *Prdm16* downregulated, in *Hes*-null forebrain. Coronal sections of wildtype (A, C, E) and *Hes cTKO* (B, D, F) E14.5 telencephalon with ISH to detect *Prdm8* (A, B), *Ngn2* (C, D), and *Prdm16* (E, F). *Prdm8* and *Ngn2* are upregulated in developing *Hes*-null brains. This upregulation is due to more cells expressing *Prdm8* (compare A′ and B′, which are magnified regions of the boxed areas in A and B respectively) and *Ngn2* (compare the brackets in C and D). On the other hand, *Prdm16* is strongly downregulated in the subpallium (blue arrowheads) and slightly downregulated in the pallium (purple arrowheads). Scale bar in A, 500 µm.

Three *Hes* genes (*Hes1*, *3* and *5*) can potentially substitute for each other in the ventricular zone [39]; hence we chose to analyze the expression of *Prdm8* and *16* in *Hes* triple-null forebrain. To obtain Hes triple-null forebrains we crossed *Hes1* (floxed/floxed); *Hes3* ^−/−^; *Hes5* ^−/−^ mice with Emx1-Cre; *Hes1* ^+/−^; *Hes3* ^−/−^; *Hes5* ^−/−^ mice. We hereafter refer to the triple-null forebrains of the embryos derived from this cross as *Hes cTKO* forebrains. We examined E14.5 *Hes cTKO* forebrains for both *Prdm8* and *Prdm16* expression as well as the expression of the proneural factor *Ngn2.* It has previously been shown that in *Hes cTKO* mutant embryos cortical neurogenesis occurs prematurely [39]. Indeed, in *Hes cTKO* mutant telencephalon, compared to wildtype, there was a large increase in the number of cells expressing *Prdm8* (Fig. 6A, A′, B, B′, n = 4) and *Ngn2* (Fig. 6C, D, bracket, n = 4). These data show that, like *Ngn2*, *Prdm8* expression is repressed by *Hes* activity, and they further imply that *Prdm8* is activated during the temporal progression of neurogenesis in a fashion similar to *Ngn2*. Furthermore, in *Hes cTKO* telencephalon the progenitor cell population is reduced but not fully depleted [39]. Indeed, in *Hes cTKO* forebrains there was also a thinning of the *Prdm16* expressing VZ region in the telencephalon (Fig. 6E, F, purple arrowheads) and a stronger reduction of *Prdm16* in the VZ of the striatum (Fig. 6E, F, blue arrowheads, n = 4).

**Figure 7:**
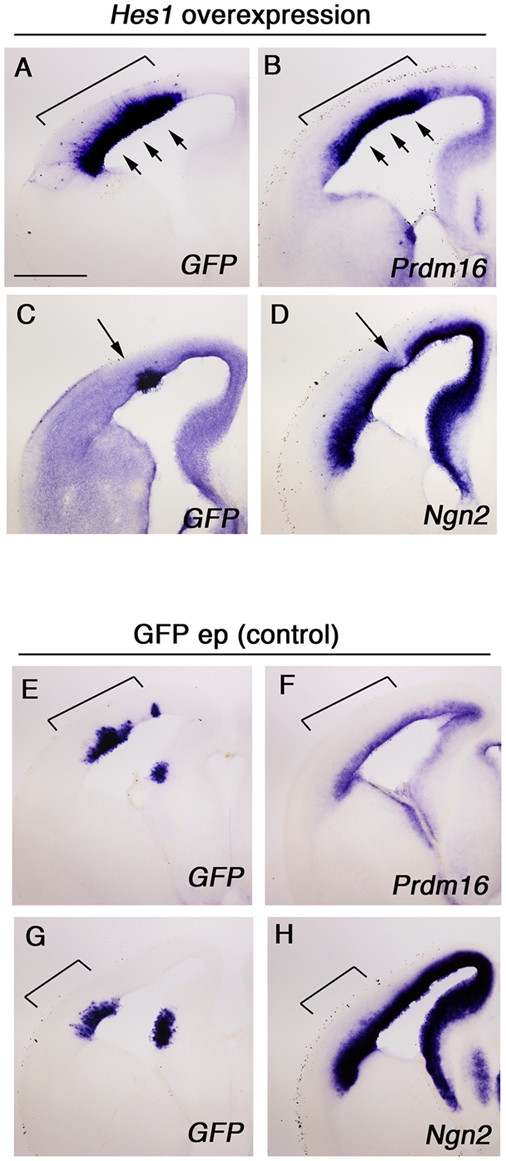
*Prdm16* is upregulated in response to *Hes1* overexpression. pEF-EGFP and pEF-Hes1 together (A–D) or a control consisting of pEF-EGFP alone (E–H) were electroporated into telencephalic neural progenitors at E13.5. Eighteen hours later, the brains were harvested, sectioned coronally, and then ISH was carried out to detect *GFP* (A, C, E, G), *Prdm16* (B, F), and *Ngn2* (D, H). *Hes1* electroporation into the telencephalon caused upregulation of *Prdm16* and a concomitant downregulation of *Ngn2*. Scale bar in A, 500 µm.

*Hes1* promotes neural progenitor cell identity by repressing genes that promote cell cycle exit and neural differentiation [38]. Targets of *Hes1* activity can be determined by *Hes1* electroporation into the telencephalon followed by examination of changes in putative target gene expression via ISH [38]. Our *Hes* loss of function (*Hes cTKO*) data implied that *Prdm16* may be positively regulated by *Hes1*, and hence be part of a suite of genes expressed in neural progenitor cells. To examine if *Prdm16* is positively regulated by *Hes* we performed *in utero* electroporation of *Hes1* cDNA along with *EGFP* cDNA (pEF-Hes1 and pEF-EGFP) into the telencephalon at E13.5 (Fig. 7A–D); we also electroporated *EGFP* cDNA alone as a control (Fig.7E–H, bracket, n = 5, respectively). Eighteen hours later we sacrificed the embryos and carried out ISH to determine *Prdm16* gene expression. *Prdm16* was strongly upregulated by *Hes1* overexpression (Fig. 7A, B, bracket and arrowheads, n = 5) implying that *Prdm16* is positively regulated by *Hes1* during neurogenesis and expressed in the neural progenitor cell population. As Hes1 protein is believed to act as a transcriptional repressor [3] positive regulation of *Prdm16* by *Hes1* may not be direct; it is possible that *Hes1* acts by repressing a repressor of *Prdm16* expression. At the same time we examined *Ngn2* expression, and as previously reported *Ngn2* was repressed by *Hes1* overexpression (Fig. 7C, D, arrow, n = 4) [38]. Electroporation of *EGFP* alone did not cause any change in *Prdm16* or *Ngn2* gene expression (Fig. 7E–H).

## Discussion

### Prdm family-mediated neural class specification of the developing spinal cord

Our data suggest that several members of the *Prdm* family could play a role in neuronal specification. During spinal cord development distinct classes of neurons are generated from progenitor cells located at different dorso-ventral positions within the VZ. These domains are spatially defined by the restricted expression of members of the HD transcription factor family and the bHLH factor *Olig2*. The individual code of transcription factors expressed in each region of VZ defines the fate of the neurons that are generated in that specific region [32]. We can now add the *Prdm* transcription factors as another family in which multiple members delineate specific progenitor regions. The *Prdm* family is hence an interesting candidate for involvement in controlling neuron class identity in the spinal cord.

HD transcription factor proteins expressed in the spinal cord VZ are divided into two groups, classes I and II. A single class I and a single class II factor are paired in such a way that there is a sharp boundary between the domains that express each member of the pair. HD transcription factors are repressors, and the sharp boundaries of expression between each pair of class I and II factors are achieved by mutual cross-repression [32]. Interestingly, Prdm proteins associate with a wide range of chromatin-remodelling enzymes and also act predominantly as transcription repressors [16], [40]–[44]. The expression domains of *Prdm13* and *8* have a sharp mutual border, raising the interesting possibility that these factors may repress each other. In addition, *Prdm13*, *8* and *12* all have sharp borders with domains that express specific HD factors, raising the possibility of repression between Prdm and HD factors.

In addition to progenitor cell regions, *Prdm8* and *12* are expressed in adjacent cells in the mantle zone. Furthermore, both *Prdm8* and *13* are expressed at a high level by cells at the margin of the VZ. Therefore a second point in spinal cord neurogenesis that *Prdm* genes may be active is as the cells exit the proliferative zone and begin to differentiate as neurons. In this context, we note that in the *Drosophila* PNS *hamlet* specifies neuron class fate by acting only transiently at the point where intermediate precursor cells undergo a final division and the immature neuron is formed [6]. *prdm1* also acts in a transient fashion to specify slow twitch muscle in zebrafish [19]. It is tempting to speculate that such transient Prdm protein activity could involve establishing a new stable chromatin state in the differentiating cells, either by direct PR domain-mediated remodelling or indirectly by recruiting remodelling enzymes [16], [40]–[44].

### Prdm family-mediated patterning of the developing brain

In the developing telencephalon patterning is controlled by transcription factors expressed not in discrete domains but rather in a graded fashion [45]. In the telencephalon both *Prdm12* and *16* are expressed in lateral/strong to medial/weak gradients (Fig. 4). Furthermore, initial domains of *Prdm12* expression are adjacent to the Zli and isthmus, both of which act as signalling centres regionally patterning the developing brain. Hence, *Prdm16* and especially *Prdm12* are candidates that merit further examination for roles in brain patterning.

In the early postnatal brain *Prdm8* is expressed in cortical layers 2/3 and 4, and *Prdm12* in the hippocampus, part of the hypothalamus, and the thalamus (Fig. 5). Furthermore, outside the brain, *Prdm12* is strongly expressed in both dorsal root and cranial ganglia. These expression domains imply that, in addition to a possible involvement in patterning, these factors may also play a role in the differentiation and function of specific neuron classes.

### Probable evolutionary conservation of individual Prdm family member functions during vertebrate neurogenesis

A recent survey in zebrafish described homologues of the entire mouse *Prdm* gene family [46]. Zebrafish *Prdm* family members that have CNS specific expression during neurogenesis (*prdm8a*, *8b*, *12*, *13* and *16*) are the homologues of those mouse *Prdm* family members we describe in this study [46]. Furthermore, there is considerable conservation in the domains of expression for some of these homologues, suggesting probable evolutionary conservation of function. For example, zebrafish *prdm8a* and *13* are expressed in the spinal cord [46] similar to *Prdm8* and *13* in mouse and it will be interesting to ascertain if they have analogous expression domains along the dorso-ventral axis. In the developing telencephalon, similar to mouse, zebrafish *prdm16* is expressed during early neurogenesis (18 hours postfertilization) and downregulated later (by 24 hours postfertilization) [46]. These data suggest an early role for *prdm16* in telencephalic neurogenesis and raise the question of whether *prdm16* also marks neural precursors in zebrafish as it does in mouse. *prdm3* is also expressed in early telencephalic development in zebrafish [46] but not mouse ([23] and data not shown); this overlapping telencephalic expression of *prdm3* and *16* in zebrafish is interesting because they encode very similar proteins that are likely to share conserved mechanisms of action [40]–[42].

### Putative conserved roles of Prdm family members in neuronal, smooth muscle, germ cell, and haemopoietic development, and in leukaemogenesis

*Prdm16*, originally named *Mel1*, was first identified as being expressed at highly elevated levels in leukaemia. Expression of high levels of a truncated form of *Prdm16* that does not encode the PR domain (called *sPrdm16* or *Mel1s*) is associated with acute myeloid leukaemia (AML) in humans [11], [47] and causative of AML in mouse [48], [49]. In this study we demonstrated that *Prdm16* expression in the developing CNS is mediated by the Notch-Hes pathway. Interestingly, haemopoiesis also utilizes Notch signalling to maintain stem cell identity and to diversify cell types during lineage elaboration [50]. Moreover, certain mutations that constitutively activate Notch1 protein cause leukaemia; although this is usually T-cell acute lymphoblastic leukaemia rather than the AML associated with *Prdm16* [51]. Hence, the relationship of *Prdm16* to Notch signalling and a conserved role for *Prdm16* in the maintenance of progenitor cell fate are very interesting prospects for further investigation in haemopoiesis and leukaemogenesis, as well as neurogenesis.

Intriguingly, *Prdm3*, originally named *Mds1/Evi1*, is the *Prdm* family member most closely related to *Prdm16* [8]. *Prdm3* also causes AML when a truncated form (called *Evi1*) that does not encode the PR domain is ectopically expressed [10], [52]. Although *Prdm3* is not expressed in developing mouse telencephalon ([23] and data not shown), it is expressed in embryonic and adult haemopoietic progenitors in which it regulates proliferation [53]. The domain structure of the Prdm16 and Prdm3 proteins are very similar (Fig. 1) and both regulate transcription through binding to the same co-factors, in particular the co-repressor C-terminal Binding Protein (CtBP) [40]–[42]. These results suggest that Prdm16 and Prdm3 proteins could function in neural progenitor cells, haemopoietic progenitors, and oncogenic haemopoietic progenitors via closely related mechanisms. Hence, previous studies of Prdm3 function in haemopoiesis and leukaemogenesis may be relevant to Prdm16 function in progenitor cells in the CNS.

Other *Prdm* family members also play roles in maintaining precursor cell proliferation and pluripotency. *Prdm6* is expressed in a variety of smooth muscle-containing tissues where it acts to suppress differentiation and maintain the proliferative potential of vascular smooth muscle precursors [16]. Pluripotency is an essential feature of germ cells, and a recent report by Saitou and colleagues has described a two-step process in germ cell specification that involves the sequential activity of two *Prdm* family members [54]. First *Prdm1* acts to repress the somatic gene expression program; second *Prdm14* acts to promote the reacquisition of pluripotency and genome-wide epigenetic reprogramming. Notably, *Prdm14* is also upregulated in human ES cells where it suppresses differentiation [55]. An interesting potential link between *Prdm14* function and *Prdm16* is that *Prdm14* mediates the acquisition of germ cell pluripotency in part by upregulating *Sox2* [54]. *Sox2* also has a crucial role in neural precursor cell proliferation and maintenance [56]; hence in addition to the *Hes* factors, *Sox2* is a very good candidate for interaction with *Prdm16* during neurogenesis.

### Conclusions

In this study we have shown that several members of the *Prdm* gene family (*Prdm6*, *8, 12, 13* and *16*) have interrelated expression patterns during mouse CNS neurogenesis, which suggest roles in neuronal class specification and differentiation. Within the telencephalon we find that *Prdm16* marks neuronal progenitor cells and *Prdm8* postmitotic neurons. In this brain region *Prdm16* expression is maintained by Notch-Hes signalling and transition to *Prdm8* expression follows the down regulation of Notch. This relationship between *Prdm* genes and the Notch-Hes pathway will be interesting to investigate in wider developmental and oncogenic contexts. Interestingly, our study and a very recent study both show conservation of *Prdm16* interaction with bHLH factors; *Prdm16* interacts with *Hes1* in this study and with *Myf5* in the skeletal muscle to brown fat fate switch [18]. It is now important to ascertain if there are common mechanisms of *Prdm16* (and *Prdm3*) interaction with the Notch pathway during brown fat determination, neurogenesis, haemopoiesis, and leukaemogenesis. Certainly, the data we present in this study show that the *Prdm* family interacts with the Notch-Hes pathway during neurogenesis, may control nervous system patterning, and may modulate neuronal progenitor cell proliferation and differentiation. Hence, the *Prdm* family is an excellent candidate for further investigation relating to the generation of nervous system complexity.

## Materials and Methods

### Sequence analysis

The nucleotide and peptide sequences used for primer design to generate ISH probes from *Prdm* gene family members were obtained from the NCBI and MGI databases. The accession numbers of the cDNAs or predicted gene sequences used for the design of the primers used in this study were as follows: *Prdm5*, NP_081823; *Prdm6*, NP_001028453; *Prdm8*, NP_084223; *Prdm9*, XP_619431.3; *Prdm10*, NP_001074286; *Prdm11*, CAM14371; *Prdm12*, XM_355325.5; *Prdm13*, NP_001074240; *Prdm14*, NP_001074678; *Prdm15*, XP_622716; *Prdm16*, NP_081780. All plasmids used to generate probes for ISH are freely available upon request from A.W.M. Protein domains for Figure 1 were identified by utilizing PFAM [57] and BLAST.

### Molecular biology

Total RNA was prepared from ICR mouse embryos at E11.5 (whole embryos) and E13.5 (head) using an RNeasy Mini Kit (QIAGEN). RT-PCR was performed using a OneStep RT-PCR kit (QIAGEN) as per manufacturer's instructions. The individual PCR products were cloned into the plasmid vector pGEM-T Easy (Promega). Additional probes used were: *Hes5*, *classIII ß-tubulin*, *Ngn2, Dbx1*, *Olig2, Nkx2.2, Tbr1*, and *GFP*. Both whole mount and section mRNA ISH were carried out using previously published one- or two-colour methods [58].

### Mouse breeding

*Hes* mutant mice were generated as described previously [39]. *In utero* electroporation experiments were designed and executed as described in recently published studies [38], [59]. All animal research and husbandry was completed in accordance with the guidelines of the RIKEN Brain Science Institute and Kyoto University.

## Supporting Information

Table S1 Sequence analysis that indicates the position of protein domains for Prdm1-16.
(0.04 MB DOC)
