# Supplementary material for: Prdm Proto-Oncogene Transcription Factor Family Expression and Interaction with the Notch-Hes Pathway in Mouse Neurogenesis
Source: PLoS One. 2008 Dec 3;3(12):e3859. doi: 10.1371/journal.pone.0003859 (PMC2585159; doi:10.1371/journal.pone.0003859)
Supplement: Table S1 — Sequence analysis that indicates the position of protein domains for Prdm1-16. (0.04 MB DOC) [file pone.0003859.s001.doc]

| Protein | Database Identifier | Size  (amino acids) | PR domain | Zinc Finger(s) |
| --- | --- | --- | --- | --- |
| Prdm1 | NP_031574 | 823 | 77-219 | 573-595  601-623  629-651  657-679 |
| Prdm2 | NP_001074824 | 1700 | 21-135 | 347-369  377-399  468-491  1174-1197  1315-1337  1437-1460 |
| Prdm3 | not available | 1232 | 74-212 | 211-234  265–287  293–315  321–344  350–372  378–400  407–429  914–936  942–965  971–993 |
| Prdm4 | NP_857633 | 803 | 402-551 | 593-615  621-642  649-671  677-699  705-727  733-755 |
| Prdm5 | NP_081823 | 599 | 2-143 | 167-190  199-221  231-256  264-286  289-311  317-339  345-367  373-395  401–242  430-452  458-480  486-508  514-536  542-564  571-594 |
| Prdm6 | NP_001028453 | 596 | 241-365 | 474-496  502-524  530-552  558-583 |
| Prdm8 | NP_084223 | 688 | 7-144 | 665-687 |
| Prdm9 | XP_619431.3 | 843 | 238-377 | 537-559  565–587  593–615  621–643  649–671  677–699  705–727  733–755  761–783  789–811  817–839 |
| Prdm10 | NP_001074286 | 1135 | 149-269 | 300-322  471-493  501-523  529-551  557-580  585-607  613-636  668-691 |
| Prdm11 | CAM14371 | 565 | 109-245 | none |
| Prdm12 | XM_355325.5 | 365 | 80-222 | 242-264  270-292  298-322 |
| Prdm13 | NP_001074240 | 754 | 62-179 | 185-207  620-642  648-670  677-700 |
| Prdm14 | NP_001074678 | 561 | 235-346 | 390-414  422-445  451-473  479-501  507-530  536-558 |
| Prdm15 | XP_622716 | 1174 | 69-204 | 402-424  434-457  495-517  522-544  571-593  598-620  661-684  689-711  725-747  753-775  781-803  809-831  837-859  865-88  894-917 |
| Prdm16 | NP_081780 | 1275 | 76-226 | 282-304  310 -332  338-361  367-389  395-417  424-446  951-971  979-1102  1008-1030 |
